# Editorial: Gasdermins in the defense against pathogens

**DOI:** 10.3389/fimmu.2023.1238368

**Published:** 2023-06-20

**Authors:** Leopold Eckhart, Ruochan Chen

**Affiliations:** ^1^ Department of Dermatology, Medical University of Vienna, Vienna, Austria; ^2^ Hunan Provincial Key Laboratory of Viral Hepatitis, Xiangya Hospital, Central South University, Changsha, China

**Keywords:** innate immunity, GSDMA, GSDMD, caspase, pyroptosis, cell death, proteolysis, infection

Gasdermins are pore-forming proteins that are activated by proteolytic cleavage (1). Processing of gasdermins by caspase-1 or caspase-4/5/11 separates the carboxy-terminal repressor domain from the amino-terminal domain which oligomerizes and inserts into membranes to form a pore. Gasdermin-mediated perforation of the plasma membrane leads to the secretion of proinflammatory molecules and cell death, known as pyroptosis (2). The identification of gasdermin homologs in prokaryotes and their implication in the protection against phages indicate an evolutionary ancient role of gasdermins in host defense against infections (3). Recent research has revealed that mammalian gasdermin A (GSDMA) can be activated by bacterial proteases to trigger pyroptosis (4, 5), indicating a dual function of gasdermins as direct sensors of pathogens and executors of an immediate host response that limits the spread of infections.

The aim of this Research Topic is to explore the roles of gasdermins in host-pathogen interactions at the molecular level. Original research articles and reviews address different roles of gasdermins in infections with pathogens. The focus of the Research Topic is on the contributions of gasdermins to antimicrobial defense and their relevance for infectious diseases.

Li et al. review the functions and regulation of gasdermin D (GSDMD) in pyroptosis and highlight its importance for a wide range of diseases. Potential strategies for the therapeutic targeting of GSDMD and particularly inhibitors of GSDMD-mediated pore formation are discussed.

Greenwood et al. focus on the control of GSDMD by metabolic processes and autophagy, and extend the scope of their review to all members of the gasdermin family. Furthermore, they discuss the challenges faced during the pharmacological development GSDM inhibitors. This article reviews the state-of-the-art and potential future directions of this promising field of drug research.

The critical role of gasdermins in pyroptosis is explored in the paper by Pan et al. This reviews shows a timeline of the discoveries that were key to the astonishing progress in this field of research. Importantly, different caspases are activated in response to infection with different bacteria and in pathological settings, suggesting that these caspases and the canonical or non-canonical activation of gasdermins may be specific targets for interventions to control the course of diseases.

Liang et al. report the induction of GSDMD-mediated pyroptosis by pyolysin, a cholesterol-dependent pore-forming toxin that is secreted by *Trueperella pyogenes*, a gram-positive bacterium causing mastitis in cows and various other infectious diseases in diverse species. In this setting, pores formed by pyolysin cause the efflux of potassium ions, leading to the activation of the NLRP3 inflammasome and the subsequent formation of GSDMD pores through which interleukin (IL)-1 beta is released.

Lee et al. investigated the activation of GSDMD in response to cold-inducible RNA-binding protein (CIRP), which is released from its normal nuclear localization during sepsis and trauma. CIRP is recognized by macrophages as a damage-associated molecular pattern (DAMP) and triggers the release of DNA, nuclear and cytoplasmic proteins which form so-called extracellular traps for pathogens. Macrophage extracellular traps (METs) are equivalent to the more widely known neutrophil extracellular traps (NETs) (6). The formation of METs was blocked by an inhibitor of caspase-1 and by disulfiram, an inhibitor of GSDMD (7).

The involvement of GSDMD in the secretion of IL-36γ is suggested by Manzanares-Meza et al. Necrosulfonamide, a chemical that binds to proteolytically processed GSDMD and prevents pore formation (8), reduced the release of IL-36γ, a member of the IL-1 family of cytokines, from a murine macrophage cell line. The results of this study will stimulate further investigations in other cell types, possibly involving targeted deletion of the *GSDMD* gene.

Chen et al. report that a cell-permeable small molecule, called JQ1 (9), which inhibits bromodomain and extra-terminal family protein BRD4, reduced the level of NF-κB phosphorylation, inflammasome formation and processing of GSDMD in an experimental model of endotoxemia of the colon.

The articles of this Research Topic highlight gasdermins as regulators and executors of innate immunity. An improved understanding of gasdermin-dependent processes is the basis for the definition of gasdermins as a biomarkers and for the pharmacological targeting of either upstream regulators of gasdermins or gasdermins themselves. Given the important roles of gasdermins in combating infections, it is important to aim for the specific correction of gasdermin dysregulation without disrupting essential innate immune responses. Although many aspects of gasdermins require further research, the articles of this compilation provide a valuable perspective on the translation of insights from basic science into the therapeutic targeting of gasdermins for improved anti-microbial defense and suppression of undue activation of gasdermins in diseases.

## Author contributions

All authors listed have made a substantial, direct, and intellectual contribution to the work and approved it for publication.
